# Supplementary material for: Role and mechanism of NCAPD3 in promoting malignant behaviors in gastric cancer
Source: Front Pharmacol. 2024 Apr 22;15:1341039. doi: 10.3389/fphar.2024.1341039 (PMC11070777; doi:10.3389/fphar.2024.1341039)
Supplement: Supplementary file 11 [file DataSheet2.ZIP › GSEA/Oncogenic signature/my_analysis.Gsea.1599461962066/P53_DN.V1_UP.html]

Details for gene set P53\_DN.V1\_UP[GSEA]

|  || Dataset | filtered\_dataset.sample\_info.cls#WT\_versus\_NCAPD3\_MUT |
| Phenotype | sample\_info.cls#WT\_versus\_NCAPD3\_MUT |
| Upregulated in class | NCAPD3\_MUT |
| GeneSet | P53\_DN.V1\_UP |
| Enrichment Score (ES) | -0.44161597 |
| Normalized Enrichment Score (NES) | -2.3503025 |
| Nominal p-value | 0.0 |
| FDR q-value | 0.003585157 |
| FWER p-Value | 0.007 |
Table: GSEA Results Summary

  

Fig 1: Enrichment plot: P53\_DN.V1\_UP      
 Profile of the Running ES Score & Positions of GeneSet Members on the Rank Ordered List

  

| SYMBOL | TITLE | RANK IN GENE LIST | RANK METRIC SCORE | RUNNING ES | CORE ENRICHMENT || 1 | 323 | APBB2 | 35 | 0.977 | 0.0347 | No |
| 2 | 7464 | CORO2A | 138 | 0.757 | 0.0072 | No |
| 3 | 4091 | SMAD6 | 519 | 0.462 | -0.2399 | No |
| 4 | 1119 | CHKA | 670 | 0.390 | -0.3247 | No |
| 5 | 4507 | MTAP | 690 | 0.384 | -0.3149 | No |
| 6 | 3673 | ITGA2 | 858 | -0.264 | -0.4198 | No |
| 7 | 6385 | SDC4 | 871 | -0.282 | -0.4112 | No |
| 8 | 301 | ANXA1 | 914 | -0.342 | -0.4206 | Yes |
| 9 | 9414 | TJP2 | 916 | -0.345 | -0.4001 | Yes |
| 10 | 3913 | LAMB2 | 919 | -0.348 | -0.3801 | Yes |
| 11 | 10232 | MSLN | 925 | -0.351 | -0.3622 | Yes |
| 12 | 3691 | ITGB4 | 926 | -0.352 | -0.3405 | Yes |
| 13 | 4052 | LTBP1 | 928 | -0.353 | -0.3196 | Yes |
| 14 | 3489 | IGFBP6 | 934 | -0.359 | -0.3011 | Yes |
| 15 | 725 | C4BPB | 950 | -0.368 | -0.2894 | Yes |
| 16 | 4680 | CEACAM6 | 955 | -0.374 | -0.2693 | Yes |
| 17 | 7277 | TUBA4A | 997 | -0.404 | -0.2742 | Yes |
| 18 | 6286 | S100P | 1011 | -0.412 | -0.2583 | Yes |
| 19 | 23764 | MAFF | 1039 | -0.436 | -0.2511 | Yes |
| 20 | 1308 | COL17A1 | 1065 | -0.455 | -0.2413 | Yes |
| 21 | 5268 | SERPINB5 | 1089 | -0.467 | -0.2293 | Yes |
| 22 | 26053 | AUTS2 | 1113 | -0.485 | -0.2161 | Yes |
| 23 | 1032 | CDKN2D | 1156 | -0.519 | -0.2147 | Yes |
| 24 | 27074 | LAMP3 | 1158 | -0.522 | -0.1833 | Yes |
| 25 | 2152 | F3 | 1188 | -0.559 | -0.1700 | Yes |
| 26 | 6038 | RNASE4 | 1194 | -0.570 | -0.1386 | Yes |
| 27 | 3866 | KRT15 | 1214 | -0.589 | -0.1162 | Yes |
| 28 | 8412 | BCAR3 | 1261 | -0.648 | -0.1097 | Yes |
| 29 | 1956 | EGFR | 1266 | -0.657 | -0.0722 | Yes |
| 30 | 202 | AIM1 | 1290 | -0.681 | -0.0470 | Yes |
| 31 | 5794 | PTPRH | 1368 | -0.854 | -0.0504 | Yes |
| 32 | 4312 | MMP1 | 1407 | -1.304 | 0.0022 | Yes |
Table: GSEA details [plain text format]

  

Fig 2: P53\_DN.V1\_UP      
 Blue-Pink O' Gram in the Space of the Analyzed GeneSet

  

Fig 3: P53\_DN.V1\_UP: Random ES distribution      
 Gene set null distribution of ES for **P53\_DN.V1\_UP**
